# Multidisciplinary Approach to Child and Adolescent Depression

**DOI:** 10.1155/2011/854594

**Published:** 2011-08-23

**Authors:** Bettina F. Piko, Robert Milin, Rory O'Connor, Michael Sawyer

**Affiliations:** ^1^Department of Behavioral Sciences, Faculty of General Medicine, University of Szeged, Szeged, Hungary; ^2^Royal Ottawa Mental Health Centre, Institute of Mental Health Research and Department of Psychiatry, University of Ottawa, Ottawa, Canada; ^3^Department of Psychology, University of Stirling, Stirling, Scotland, UK; ^4^Research and Evaluation Unit, Women's and Children's Hospital and School of Paediatrics and Reproductive Health, University of Adelaide, Adelaide, Australia

In collaboration with a qualified international scientific team, we are pleased to launch this special issue which contains papers from epidemiological aspects to clinical implications with special emphasis put on psychological models and theories. 

 The special issue on child and adolescent depression is also an arena for multidisciplinarity and covers a wide range of viewpoints and contexts. Hopefully, with this volume, we provide a platform for a need of subsequent communication between basic sciences and clinical experiences as the most significant challenge of the 21st medical research. This is particularly true in case of depression research where integration of basic biological sciences with possible application areas is fundamental.

Depression contributes to morbidity and mortality across the life course; therefore it is particularly important to detect influences of depressive symptoms in childhood since these may have longer-term adverse effects on the psychosocial adjustment of adults [1]. The frequency of depressive symptoms increases markedly in adolescence [2]. Both biological-hormonal changes and environmental factors are likely to contribute to this increase [3]. As research results suggest, early adolescence is a time when the prevalence of depressive symptoms increases markedly, particularly among females [4, 5]. This is due to intensified gender socialization in this age period [6].

Not surprisingly, adolescent depression has become a focus in current scientific research. However, there is evidence that depressive symptoms tend to begin before puberty. As a result, more attention should be paid to depression in children and the preadolescent period as well. Unfortunately, we know much less about the contextual factors of child and adolescent depression. We need more research into different aspects of this phenomenon including a multidisciplinary/biopsychosocial approach. Critical evaluations and review articles are particularly useful for integrating empirical research results. Early detections and interventions of mood disorders are perhaps a greatest challenge for preventive psychiatry and mental health promotion. As a consequence, we have lots of undiscovered fields in child and adolescent psychiatry and psychology related to child and adolescent depression. Apart from this editorial, a total of 11 articles are offered to the readers in this issue; most of them are theoretically based research papers with two reviews and studies on psychological models, comorbid states, and prevention issues. 

Longitudinal studies are particularly relevant for investigating the role of psychological models which may help us understand underlying mechanisms of how adolescent depression may develop. In a longitudinal study, Z. Gutkovich and his colleagues investigated the role of anhedonia and pessimistic attributional style in a sample of hospitalized depressed adolescents. Anhedonia (the diminished capacity to experience pleasure) and pessimistic attributional style (the manner in which a person explains the cause of an event in a negative way) are of special interest in relation to major depression. As the authors found, anhedonia is a critical characteristic of adolescent depression, one that could be intimately involved in the pathogenic mechanism of the depressive episode through association with pessimistic attributional style. 

In the next paper, published by A. H. Mezulis et al., prospective associations between negative emotionality, rumination, and depressive symptoms was examined in a community sample of youth followed longitudinally from birth to adolescence. The authors applied a cognitive model of depression where ruminative response style represented a cognitive vulnerability. Results found that greater negative emotionality in infancy was associated with more depressive symptoms at age 15, and rumination significantly mediated the association between them for girls but not for boys.

The third longitudinal study, carried out by Y. M. Sanchez and coworkers, aimed to investigate association between adverse life events and depressive symptoms in African American youth. In addition, control-related beliefs were added as a mechanism linking life event stress and depression which had received little attention before. Findings suggest that control-related beliefs mediated the association between adverse life events and depressive symptoms, and a significant adverse life events → control-related beliefs → depressive symptoms indirect effect. In addition, violent life events, rather than nonviolent events, also may have predicted depressive symptoms.

Next, in this special issue, an exciting review focuses on magnetic resonance spectroscopy. G. Kondo and his team provided a thorough and critical overview of studies in this field. They concluded that, because it offers a noninvasive and repeatable measurement of relevant *in vivo *brain chemistry, MRS has the potential to provide insights into the pathophysiology of adolescent major depression MDD, as well as the mediators and moderators of treatment response.

As it has already been noted here, preadolescent child depression is getting more and more attention. In an exciting paper of M McCabe et al., the focus has been laid to this age group and the psychosocial functioning in relation to depression. The authors concluded that it was harder for the social network of the children to detect the problems of children with elevated depressive symptoms, who did not meet the diagnostic criteria. Laypeople usually differentiated between the “clinical” and “normal” groups. The authors recommended that it might be important to implement intervention programs including “at-risk” children as well.

The next papers deal with comorbidity. L. -G. Lundh and colleagues provided longitudinal research data on depressive symptoms and deliberate self-harm in a community sample of adolescents. There was support for a bidirectional relationship between depressive symptoms and self-harm in the girls; whereas in boys, there was only support for a unidirectional relationship, depressive symptoms being a predictor of increased self-harm one year later.

In terms of comorbidity, substance use disorders occupy a special place. The next three papers, not surprisingly, deal with this type of comorbidity. H. O. Anderson and A. M. Libby reported on a study on depression with and without comorbid substance dependence among young adults. As it was concluded, frequent use of substances significantly increased the likelihood of subsequent depression with comorbid substance dependence compared to depression alone.

In the next study carried out by T. Pirkola and coworkers, the authors aimed at examining the differences between depressed psychiatric adolescent outpatients with and without cooccuring alcohol misuse in psychosocial background, clinical characteristics, and treatment received during one-year followup. Alcohol misuse well indicated family problems and had a deleterious effect on treatment attendance.

Alcohol misuse is a serious problem amongst adolescents in many countries. It is a particularly serious problem amongst indigenous communities. The study of S. H. Stewart and colleagues examined the relationship between hopelessness, depression and excessive drinking in a Canadian population of adolescents, the majority of whom were aboriginal youth. The results from the study showed a strong relationship between hopelessness and depressive symptoms. They also showed a strong relationship between depressive symptoms and “drinking to cope”. In this context, it appeared that excessive alcohol intake was being used to both reduce unpleasant feelings of depression and block pessimistic thoughts commonly associated with depression. The results from the study suggest that focusing on hopelessness, depressive symptoms and drinking to cope may be important for programs trying to reduce alcohol misuse among aboriginal youth.

Finally, the last two papers deal with prevention issues. There is limited evidence that prevention programs for depression are effective among adolescents [7]. However, given current knowledge about adolescent help seeking, it is important that prevention and early intervention programs include elements that aim at improving the quality of the social support offered by peers and family members to adolescents experiencing depressive symptoms. Teachers, family members, and peers should also be encouraged to actively engage with young adolescents experiencing depressive symptoms rather than waiting for them to initiate help seeking. This is particularly important for adolescents experiencing higher levels of depressive symptoms who may not initiate help seeking themselves. A promising approach in this area is the use of mental health first-aid programs being developed in Australia [8]. Mental health first-aid programs focus on key steps which can be taken by teachers, family members, and peers to support adolescents with depression while guiding them to professional sources of help. The first paper that is a review carried out by F. Rice and A. Rawal focused on basic research evidence from community, clinical, and high-risk populations that identified cognitive mechanisms and emotional regulation as key processes involved in the onset and maintenance of depression. Finally, C. A. McCarty et al. reported on a novel intervention design. Despite the preliminary nature of the intervention, applying positive thoughts in school-based prevention programs seemed promising.

We hope that our readers will enjoy this special issue.
